# Standing out with Professionalism: How do Students and Faculty of two different Medical Schools perceive it?

**DOI:** 10.12669/pjms.335.13432

**Published:** 2017

**Authors:** Kamran Sattar, Sue Roff, Durdana Siddiqui, Sultan Ayoub Meo

**Affiliations:** 1 Kamran Sattar, Department of Medical Education, College of Medicine, King Saud University, Riyadh, Saudi Arabia; 2 Sue Roff, Center for Medical Education, University of Dundee, Scotland, UK; 3 Durdana Siddiqui, Department of Obe Gynae, Sulaiman Al-Habib Hospital, Riyadh, Saudi Arabia; 4 Sultan Ayoub Meo, Department of Physiology, College of Medicine, King Saud University, Riyadh, Saudi Arabia

**Keywords:** Academic environment, Professionalism lapses, Recommended sanctions, Medical schools

## Abstract

**Background and Objectives:**

Medical professionalism is an essential aspect of medical education and practice worldwide. Our objective was to explore and compare the perception as recommended sanctions about professionalism lapses, using the “*Dundee Polyprofessionalism Inventory I: Academic Integrity”*, among the faculty and the students’ of two different medical schools in Saudi Arabia.

**Methods:**

Respondents from the two medical schools in Saudi Arabia, recommended sanctions for the first time, absolute lapses in academic professionalism were determined by using the “Dundee Polyprofessionalism Inventory 1: Academic Integrity”.

**Results:**

On comparing the faculty and students’ responses (from College of Medicine, King Saud University) with the published data (from another, unidentified medical school in Saudi Arabia) we found alignments in recommending sanctions for 14 (46.66%) behaviours among faculty and again concerning the11(36.66%) behaviours among the students of both cohorts.

**Conclusion:**

The results can be used to emphasise on the improved teaching and learning strategies in undergraduate medical students’ understanding of professionalism.

## INTRODUCTION

In the recent past, the professionalism has emerged as a sustained theme, yet medical educators are striving hard to get to uniformly accepted standards concerning the academic integrity. Professionalism has three essential principles, including patient welfare, patient autonomy and social justice. Today’s physicians require to act in accordance with professional standards more than ever before^1^ yet, the Arabian faculty members and students still feel that professionalism education remains a gap in formal curricula.^2^,^3^

Professionalism is inevitably associated with a society’s culture^4^ therefore, the cultural upbringing has a chief inspiration on how an individual perceives professionalism.^5^ Although there are persistent efforts in promoting professional education in medical schools across the Arab region. It is also considered indispensable to expound the current status of medical professionalism and the importance of articulating a framework, which can help all the healthcare stakeholders comprehend and upgrade their approaches to dealing with unprofessional behavior. Despite all the continuous efforts towards achieving the ideal professional academic atmosphere, there are still some concerns over academic untruthfulness such as cheating^6^, plagiarism^7^ and unprofessional behaviour^8^ seems to be common in many medical schools.^9^

Therefore, this study aimed to explore and compare the perception of professionalism among the faculty and students of two different medical schools in Saudi Arabia which may aid in the teaching and assessment of professionalism in Arabian specific cultural context.

## METHODS

This was a cross-sectional study, which took place during the academic year 2015-16 at the Department of Medical Education, College of Medicine, King Saud University (KSU), Riyadh, Saudi Arabia. The responses, collected at KSU were compared with published results from another Saudi medical school.^10^

An anonymous, self-administered, bilingual (English and Arabic), questionnaire “*Dundee Polyprofessionalism Inventory I: Academic Integrity”* with 34 survey statements^11^ was used for gathering the participants’ answers. This inventory (in English language) has been validated in the United Kingdom (UK) where data from two UK medical schools and a national reference group of medical educators validate broad areas of settlement between students and faculty on suitable endorsements and responses to lapses in professionalism at the undergraduate level.^12^

The obtained data were stored in the computers with password protection and analysed using a statistical computer program (SPSS version 21.0). Respondents were asked to respond about first time lapses in 34 types of behaviours with no mitigating circumstances by undergraduate medical students and to recommend from following list of the10 sanctions, based on a report:^13^

- Ignore (None)
- Reprimand (verbal warning)
- Reprimand (written warning)
- Reprimand, plus mandatory counselling
- Reprimand, counselling, extra work assignment
- Failure of specific class/remedial work to gain credit
- Failure of specific year (repetition allowed)
- Expulsion from college (readmission after one year possible)
- Expulsion from college (no chance for readmission)
- Report to a regulatory body.

### Participants from KSU

1431 medical students were contacted and initially 753 responded, but three students declined to participate in the study: response rate was 52%. Out of 750, one hundred and sixty-two (22%) were first-year medical students; 195 (26%) second-year; 160 (21%) third-year; 114 (15%) fourth-year; and 122 (16%) fifth- year students. Of the total agreed participants were (n= 750), there were 441 (58.57%) males and 311 (41.30%) females and 1 (0.1) preferred not to say. There were 166 (22%) students from 17-19 years of age, 518 (68.8%) from 20-24, 69 (9.2%) from 25 or over. Out of total50 participating faculty members, 32 (64%) were male and 18 (36%) were female. 31 (62%) were aged 31-50; 18 (36%) were 51-65 and 1 (2%) were over 65 years. 21 (42%) were primarily clinical faculty members and 29 (58%) were basic medical sciences faculty members.

### Participants from another Saudi Medical School

From 103 respondents 8 (7.76%) were aged 17–19 years and 95 (92%) were aged 20–24 years. 50 (49%) of the respondents were female and 52 (51%) were male and one student opted for ‘not to say’. Two percent of the respondents were in their first year; 33 (32%) in the second year; 33 (32%) in the third; 32 (31%) in the fourth and 3 (3%) in the fifth year of the course. Of the total 64 participating faculty members, 25 (39%) were male, 36 (56%) were female and 3 (5%) preferred not to give their gender information. 14 (22%) were aged 30 years or under; 40 (63%) were aged 31–50 years; 6 (9%) were aged 51–65 and 4 (6%) were aged over 65 years. 33 (52%) were doctors and the rest were from other health professions. 47 (73%) were primarily clinical teachers and the remainder non-clinical.

## RESULTS

Our study compared the median recommended sanctions by the participants from College of Medicine, KSU and from another Saudi medical school.^10^

The sanction Reprimand, plus mandatory counselling as a median recommended sanction was selected from both the groups of KSU and another medical school of Saudi Arabia for a survey statement number (SSN) Table-I.2. The sanction “Reprimand, plus mandatory counselling” was recommended for the SSN 9. For the SSN 10, the median recommended sanction for both groups of respondents was “Reprimand, counselling, extra work assignment”.

**Table-I:** Response (as medians) similarity among FACULTY and STUDENTS of both medical schools.

*SSN	*Survey statement*	*KSU Faculty n= 50*	*Another Saudi (Babelli et al. 2015) School Students (n=753)*	*KSU Faculty n=64*	*Another Saudi (Babelli et al. 2015) School Students (n=103)*
2.	Removing an assigned reference from a shelf in the library in order to prevent other students from gaining access to the information in it	4	4	4	4
9.	Failure to follow proper infection control procedures	4	4	4	4
10.	Threatening or verbally abusing a university employee or fellow student	5	5	5	5

* SSN: Survey Statement Number

The responses comparison from faculty of both medical schools in Table-II shows that the faculty at both Medical Schools recommended “Reprimand, counselling, extra work assignment for SSN 7 & 24. Additionally, the faculty at both Medical Schools recommended “Reprimand (written warning)”, “Failure of specific class/remedial work to gain credit” and “Reprimand, plus mandatory counselling” respectively, for the SSNs 3, 6 & 27. The trend of similar responses persisted when the recommended sanction was noted to be “Reprimand (verbal warning)” and “Reprimand (written warning)” respectively, for the two SSNs i.e. 1 & 5. There was also congruence between the faculties in recommending “Failure of specific class/remedial work to gain credit” as the sanction for SSNs 4 & 8. For the SSN 11the sanction recommended by both sets of faculty was “Failure of specific year (repetition allowed)”. Additionally, “Failure of specific class/remedial work to gain credit” was the recommended sanction for SSNs 4, 6, 8 & 25.

**Table-II:** Response (as medians) similarity among the FACULTY of both Medical Schools.

*SSN	*Survey statement*	*KSU Faculty (n= 50)*	*Another Saudi (Babelli et al. 2015) School Faculty (n= 64)*
1.	Getting or giving help for course work, against a teacher’s rules (e.g. lending work to another student to look at)	2	2
3.	Signing attendance sheets for absent friends, or asking classmates to sign attendance sheets for you in labs or lectures	3	3
5.	Exchanging information about an exam before it has been taken (e.g. OSCE)	3	3
27.	Cutting and pasting or paraphrasing material without acknowledging the source	4	4
7.	Claiming collaborative work as one’s individual effort	5	5
24.	Resubmitting work previously submitted for a separate assignment or earlier degree	5	5
4.	Drinking alcohol over lunch and interviewing a patient in the afternoon	6	6
25.	Plagiarising work from a fellow student or publications/internet	6	6
8.	Altering or manipulating data (e.g. adjusting data to obtain a significant result)	6	6
6.	Forging a healthcare worker’s signature on a piece of work, patient chart, grade sheet or attendance form	6	6
11.	Attempting to use personal relationships, bribes or threats to gain academic advantages by e.g. getting advance copies of exam papers or passing exam by such pressures on staff	7	7

* SSN: Survey Statement Number

The response similarities between the two student cohorts is shown in Table-III. The students from both medical schools recommended “Reprimand, plus mandatory counselling” for the SSN 7. Interestingly, again the students at both medical schools recommended an analogous sanction i.e. “Reprimand (written warning)” for another four SSNs i.e. 1, 19, 24, 27. Both cohorts recommended “Reprimand (verbal warning)” for SSNs 3 & 17 and agreed on “Reprimand, counselling, extra work assignment” for SSN 6. KSU faculty recommended the highest median sanction (i.e. “Expulsion from college (no chance for readmission)” for the two SSNs 18 & 30, whereas, the sanction for these same two behaviours, from the faculty of the other medical school was “Report to the professional regulatory body”.

**Table-III:** Response (as medians) similarity among the STUDENTS of both Medical Schools.

*SSN*	*Survey statement*	*KSU Students (n=753)*	*Another Saudi School (Babelli et al. 2015) Students (n=103)*
3.	Signing attendance sheets for absent friends, or asking classmates to sign attendance sheets for you in labs or lectures	2	2
19.	Not doing the part assigned in group work	3	3
24.	Resubmitting work previously submitted for a separate assignment or earlier degree	3	3
27.	Cutting and pasting or paraphrasing material without acknowledging the source	3	3
1.	Getting or giving help for course work, against a teacher’s rules(e.g. lending work to another student to look at)	3	3
19.	Not doing the part assigned in group work	3	3
7.	Claiming collaborative work as one’s individual effort	4	4
6.	Forging a healthcare worker’s signature on a piece of work, patient chart, grade sheet or attendance form	5	5

## DISCUSSION

Medical professionalism is commonly described as characteristics of professional excellence, integrity and altruism.^14^ In the present study, we found alignments in recommending sanctions for 46.66% behaviours among faculty and again concerning 36.66% behaviours among the students of both cohorts. There is congruence among the faculty and students’ responses in the form of median recommended sanctions for professionalism lapses, from the two medical schools in Saudi Arabia. Numerous studies, worldwide, presented the major lapses committed by students while studying in their medical school and described that the most common expositions of academic integrity is plagiarism, impersonating a student who is absent from class, imitating signatures, gaining illegal access to examination questions, legitimizing absences by false witness or bribes, helping others to fraud in examinations, cheating in examinations, and falsifying data.^15^,^16^

The “Dundee Poly-professionalism inventory” has questions that help to bring about the perceptions of the faculty and the students in the most common areas of concern related to student fitness to practice. The General Medical Council, UK also outlines these areas as: *“criminal conviction or caution, drug or alcohol abuse, aggressive, violent or threatening behaviour, persistent inappropriate attitude or behaviour, cheating or plagiarizing, dishonesty or fraud, and unprofessional behaviour of confidentiality or attitude”*.

In the present study, we found congruence in the recommending sanctions for 14 (47%) behaviours between the two cohorts of faculty and 11 (37%) behaviours between the two cohorts of the medical students. There is also only partial congruence between the faculty and students’ responses in the form of median recommended sanctions for professionalism lapses, from the two medical schools in Saudi Arabia. These are reported in a separate paper for KSU by Babelli et al.^10^ for the other Saudi school. Although the role of faculty is of paramount importance, yet, there is a need for a unanimously accepted framework for applying professionalism attributes, where the students and the faculty, both shall agree upon the principles to be acted upon. Any educational organization where the faculty is well aware of professional responsibilities can undoubtedly support and provide opportunities for students’ professional behaviours to be promoted. Effective physician role models enable learners to internalize the principles of professionalism so that learners themselves act professionally.^17^

Medical professionalism is an essential aspect of medical education and practice worldwide and it must be adopted according to different social and cultural contexts It is also very critical for students to learn and model their professional behaviors, such as having a good attitude, empathy for the patients and also the quality of being honest.^6^ It is acknowledged through the widespread literature that there are no principal academic settings of medical professionalism currently universally applicable^5^, in turn making it very challenging to identify the reasons behind the deficiency of resemblance concerning the perception of professionalism among various respondents. Therefore, an exploration of the reasons for this relatively low lack of congruence as found in current study requires further research in the field of medical professionalism. Simmilarly, this was suggested in a previously published work,^17^ which emphasized the prominence of an obligation to tackle the challenge of teaching of professionalism to medical students as this may also help to improve a positive attitude and works as a deterrent of risky youth behaviour. Kenny et al.^18^ addressed the same challenge as he mentioned, that establishing technically capable, professional, and humanistic physicians for the 21st century is no easy task. Mountains of biomedical knowledge must be acquired, diagnostic skill attained, effective communication skills established, and a solid and pertinent understanding of the practice and role of physicians in society today must be grasped.

Medical professionalism has gained global attention over the past decade, but there still remains a lack of literature on the universal applicability of the leading professionalism framework.^19^ This study proposes an approach to build a framework for medical professionalism that emphasise on the improved teaching and learning strategies in undergraduate medical students’ understanding of professionalism.

### Study limitations

The present study is limited to exploring the practicality of an online inventory to ‘map’ student and faculty understanding of the comparative importance of professional lapses through the proxy of the recommended sanctions.

## CONCLUSIONS

The present study outcomes can be used to emphasise on the improved teaching and learning strategies in undergraduate medical students’ understanding of professionalism.

## Availability of Data and Materials

The present study data cannot be shared as it may comprise recognizing information about individual contributors; therefore, it is not publicly available. Please contact the corresponding author.
